# Comparative evaluation of the wear potential of bovine enamel against hybrid and non-hybrid ceramics under abrasive and erosive conditions: An in vitro study

**DOI:** 10.34172/joddd.025.41878

**Published:** 2025-06-30

**Authors:** Alireza Dankkoub, Zahra Shooshtari, Seyedeh Fatemeh Namdar, Pegah Sadeghnezhad, Pouria Soltaninezhad, Sara Majidinia

**Affiliations:** ^1^Department of Prosthodontics, School of Dentistry,Mashhad University of Medical Sciences, Mashhad, Iran; ^2^Dental Research Center, Mashhad Dental School, Mashhad University of Medical Sciences, Mashhad, Iran; ^3^Department of Operative Dentistry, Mashhad Dental School, Mashhad University of Medical Sciences, Mashhad, Iran; ^4^Mashhad University of Medical Science, Mashhad, Iran; ^5^Cellular and Molecular Research Center, Sabzevar University of Medical Sciences, Sabzevar, Iran; ^6^Dental Materials Research Center, Mashhad University of Medical Sciences, Mashhad, Iran

**Keywords:** Ceramics, Dental enamel, Tooth abrasion, Tooth erosion, Tooth wear

## Abstract

**Background.:**

This in vitro study compared the wear response of natural enamel when opposed to hybrid and conventional dental ceramic materials under both abrasive and erosive conditions.

**Methods.:**

Eighty enamel specimens were prepared from bovine central incisors and divided into five groups based on the antagonist material used. Each group consisted of 16 specimens, with antagonists fabricated from four different aesthetic CAD/CAM block materials: VITA Enamic (VE), Lava Ultimate (LU), Lava Plus (LP), and VITA Mark II (VM), alongside natural enamel as a control. The specimens underwent 100000 wear cycles (49 N/2 Hz) under non-erosive and erosive conditions, simulating clinical scenarios. Enamel wear was quantified through weight loss measurements. Statistical analysis was conducted using two-way ANOVA and post hoc Games-Howell test, with a significance level set at α=0.05.

**Results.:**

The study demonstrated significant variations in enamel wear when opposed to different dental ceramic materials under both erosive and non-erosive conditions (*P*<0.001 for both). The VM group exhibited the highest mean enamel wear across varying pH conditions (*P*=0.0104 and *P*=0.0900). Statistically significant differences in enamel weight loss were observed among all five groups under non-erosive conditions. However, erosive wear rates differed significantly between nearly all groups, except for comparisons between LU and VE (*P*=0.271) and LP and VM (*P*=0.180). Notably, mean enamel wear values were higher when specimens were exposed to acetic acid compared to non-erosive conditions (*P*<0.001 for all groups).

**Conclusion.:**

Despite advancements in hybrid ceramic manufacturing, natural enamel wear remains significantly lower when opposed to these materials compared to conventional ceramics. Hybrid ceramics exhibited reduced wear potential compared to feldspathic and zirconia ceramics, underscoring their clinical relevance.

## Introduction

 Tooth wear has always been a complex and multifactorial phenomenon and is defined as the loss of calcified tooth tissue structure physically or chemically.^1^ Chemical, biological, and mechanical mechanisms can all simultaneously contribute to the wear process. The literature has recognized this condition as inevitable and physiologic; therefore, it is not considered an unanticipated finding over the life span of an individual.^2^

 Both clinical and experimental findings indicate that these wear processes rarely proceed solely, and tooth wear is typically the result of interaction between erosion, abrasion, and attrition processes.^3^ The degree of tooth substance wear is highly associated with the patient’s masticatory function, parafunctional habits, salivary flow, and type of restorative or prosthetic material used as antagonists.^4^ It is desirable for the restorative material to exhibit similar mechanical properties to natural enamel. Excessive wear can contribute to clinical complications such as damaged occlusal surfaces, loss of vertical occlusal dimension, tooth sensitivity and possibly pulp necrosis, masticatory impairment, and subsequent remodeling of the temporomandibular joint (TMJ) structure and at least result in esthetic compromise.^5^

 Compared to composite resins, the inorganic filler particles and organic matrix in hybrid ceramics are interlocked, reflecting enhanced physical and mechanical properties. In case of any cracks in the ceramic phase, the polymer network prevents further progression of the fracture.^6^

 The restorative material should exhibit similar mechanical properties to natural enamel.^7^ Since there is high patient demand for natural-appearing dental prosthetics, ceramic restorations have gained ever-increasing popularity over the recent decade.^8,9^ Therefore, manufacturers have adopted multiple approaches to improve ceramic material properties, especially enhancing wear resistance.^10^ Metal-free ceramic crowns with additional zirconia, alumina, and lithium disilicate-reinforced materials have been introduced as viable treatment options that provide favorable esthetic outcomes. These novel materials foster both esthetic and functional rehabilitation of the dentition.^11^

 Conventional ceramics are known for their esthetic restorations, but their rigidity and abrasive effects on the antagonist tooth have been linked to a higher incidence of failure.^12^ To address these issues, hybrid ceramics were developed to better simulate the mechanical and optical properties of natural teeth, offering reduced fragility and hardness, easier milling, and improved clinical outcomes.^13^ These materials, which combine zirconia and silica nanoceramic fillers with organic resins, exhibit lower hardness and modulus of elasticity compared to traditional ceramics, making them less abrasive and more compatible with opposing dentition.^14^ This combination provides an ideal balance of durability, aesthetics, and wear resistance.^15^

 Hybrid ceramics are superior to conventional ceramics in terms of occlusal load compensation.^13,16^ The average amount of enamel wear at occlusal contacts ranges from 20 to 40 µm per annum.^17^ Occlusal forces are dissipated throughout the polymeric structure of hybrid ceramics and, therefore, obviate functional stress concentration. Feldspathic ceramics are proven to induce greater wear in the opposing enamel.^18^ Hybrid ceramics are an increasingly popular choice in restorative dentistry due to their outstanding esthetic qualities. Additionally, research suggests that feldspathic and hybrid ceramics may be less likely to cause wear on the opposing enamel compared to other materials. This lower wear response can help preserve the natural dentition over time.^19^

 Although hybrid ceramics have long been introduced to restorative dentistry, their wear potential has not yet been thoroughly studied. Therefore, the present in vitro study compared the wear response of natural enamel when acted upon hybrid and conventional dental ceramic materials in both abrasive and erosive conditions. In the present investigation, the null hypothesis was that there is no significant difference in enamel wear when opposed to hybrid and non-hybrid ceramic under both erosive and non-erosive conditions.

## Methods

###  Specimen preparation 

**Figure 1:**
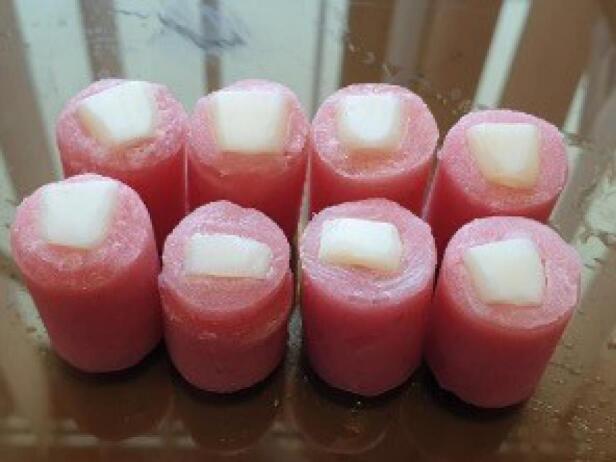
The specimens

 The protocol of this in vitro study was approved by the Research and Ethics Committee of Mashhad University of Medical Sciences (IR.MUMS.DENTISTRY.REC.1398.059). Eighty enamel specimens were prepared from the labial surfaces of 40 sound extracted bovine central incisors. Teeth with visible chipping or cracks were discarded. The teeth were sectioned in half horizontally. Two rectangular-shaped specimens (10 × 2 mm), a cervical portion and an incisal portion, were obtained from each tooth. This was achieved with manual cuts using a handpiece and diamond-coated discs. The samples were then subjected to mechanical polishing using 800–2000 grit SiC paper. The antagonists that were used in the present experiments were fabricated from four different aesthetic CAD/CAM block materials: VITA Enamic (VE), Lava Ultimate (LU), Lava Plus (LP), and VITA Mark II (VM) (Table 1). Eight #10 blocks of the aforementioned materials were sliced using a CNC machine (Nemo, Mashhad, Iran), and 16 square-shaped slices (10 × 10 × 2 mm) per material were obtained. Table 1 lists the properties of the investigated materials. The enamel specimens and ceramic antagonists were embedded into the specimen carrier using self-cured acrylic resin. The experimental groups were categorized based on the ceramic material opposed to natural enamel. Concerning the control group, bovine enamel antagonists were prepared as described above. All the samples were polished following the manufacturer’s instructions by using polishing kits. Each of the five groups consisted of 16 enamel specimens and 16 antagonists. Figure 1 shows the specimens.

**Table 1:** Properties of the utilized materials

**Group name**	**Brand**	**Material**	**N**	**Batch number**	**Manufacturer**
Enamel	-	Bovine enamel	16	Excised central incisors	Extracted teeth
VE	VITA Enamic	PICN (86% feldspathic ceramic, 14% polymer network)	16	HT-59620	VITA Zahnfabrik, Germany
LU	Lava Ultimate	Resin Nanoceramic (80% nanoceramic particles, 20% resin matrix)	16	34-8700-2348-7 HT-N420014	3 M ESPE, USA
LP	Lava Plus	Zirconium oxide ceramic	16	-	3 M ESPE, USA
VM	VITA Mark II	Feldspathic ceramic	16	2C-16,630	VITA Zahnfabrik, Germany

VE: VITA Enamic, LU: Lava Ultimate, LP: Lava plus VM: VITA Mark II.

###  Wear simulation and quantitative analysis of tooth wear

**Figure 2:**
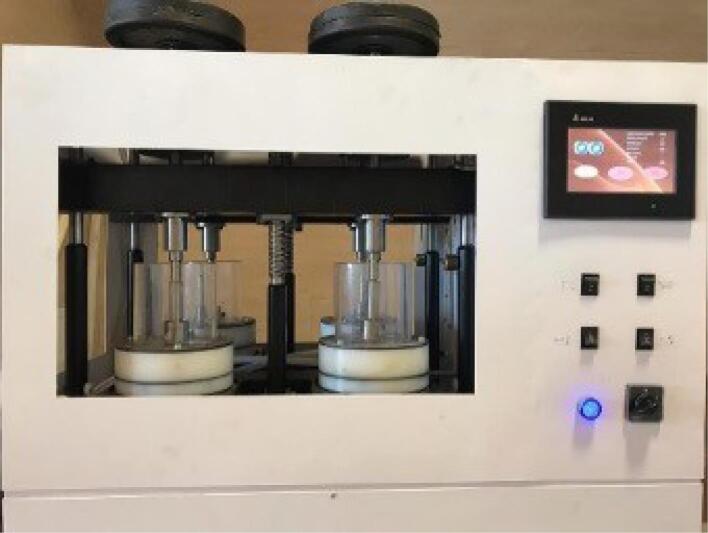
Nemo wear machine used to simulate mastication

 Nemo wear machine (Nemo, Mashhad, Iran) was used as an in vitro apparatus to simulate mastication (Figure 2). The specimens and corresponding antagonists were mounted in the wear machine and subjected to 100 000 wear cycles of bidirectional movements with a speed of 2 Hz. The enamel specimen and opposing ceramic moved laterally at a 49-N force and 15-mm sliding distance against each other. These testing conditions are believed to simulate 6 months of clinical service.^20^ Half of the test runs were conducted under artificial saliva (non-erosive conditions), and the remaining half under a demineralizing solution (erosive conditions). Throughout half of the experiments, artificial saliva (37 °C) served as a lubricant to mimic the oral environment. The artificial saliva formulation contained Ca(NO_3_)_2_4H_2_O (calcium nitrate tetrahydrate) (1 mM), NH_4_H_2_PO_4_ (ammonium dihydrogen phosphate) (3 mM), and NaCl (sodium chloride) (100 mM). The pH of artificial saliva was checked daily and adjusted to 7 with NaOH (1 mmol). To generate an erosive wear laboratory setting, specimens were immersed in acetic acid (0.125 mM) with a pH of 2.5 during the testing procedures.

 The specimens were air-dried at ambient temperature for 24 hours before final weight measurements. In this study, the rate of tooth substance wear was quantified through weight loss. Enamel specimens were weighed before and after testing using a precision balance (Denver, USA) with an accuracy of 0.1 mg. The difference between the recorded values was calculated and defined as the amount of enamel wear.^21^

###  Sample size calculation

 Based on previously published data by El Zhawi et al^22^ and according to long-term mouth-motion fatigue/wear variables, a sample size of 8 specimens per group was deemed appropriate based on the specimen capacity of the modified Alabama wear testing device. Previous studies observed the sample size of 8 per group to be sufficient for wear studies using chewing simulation under a standardized controlled environment.^23^

###  Statistical analysis

 The Kolmogorov-Smirnov test was used to test data homogeneity. The Shapiro-Wilk test revealed that the investigated variables follow a normal distribution, so parametric tests were used. Two-way analysis of variance (ANOVA) and post hoc Games-Howell statistics were employed to detect differences in the recorded wear rate values between the different materials in erosive and non-erosive conditions. Independent-samples t-test was used to compare the wear rate of bovine enamel under erosive and non-erosive conditions with respect to study groups. Data were analyzed using SPSS 23 (SPSS Inc, IBM Corp, Armonk, NY). Statistical significance was set at *P* < 0.005.

## Results

 The results of erosive and non-erosive wear tests for each study group are summarized in Table 2. Initially, a two-way ANOVA was used. However, since the interaction effect between the two factors was significant (*P* < 0.001), each factor was compared at the levels of the other factor. Given that the material factor had 5 levels and the pH factor had 2 levels, a one-way ANOVA was used to compare the material factor at each level of the pH factor. An independent t-test was applied to compare the pH factor at each level of the material factor. The bovine enamel, opposed to different materials, exhibited significantly different degrees of wear under erosive and non-erosive conditions (*P* < 0.001 for both). The VM group showed the greatest average enamel specimen wear when tested in different pH conditions (*P* = 0.01 and 0.09).

**Table 2:** Erosive and Non-erosive enamel wear rates in different test groups

**pH**	**Group**	**Number**	**Mean (mg)**	**Standard deviation**	**Min**	**Max**	**Two-way ANOVA**
7	Enamel-Enamel	8	0.0037	0.0007	0.0028	0.0049	F = 71.68 *P* < 0.001
	Enamel-LU	8	0.0070	0.0004	0.0065	0.007	
	Enamel- VE	8	0.0076	0.0007	0.0063	0.0083	
	Enamel-LP	8	0.0091	0.0004	0.0081	0.0095	
	Enamel-VM	8	0.0104	0.0015	0.0090	0.0130	
2.5	Enamel-Enamel	8	0.0537	0.0027	0.499	0.574	F = 520.63 *P* < 0.001
	Enamel-LU	8	0.0672	0.0018	0.0650	0.0699	
	Enamel- VE	8	0.0723	0.0009	0.0710	0.0735	
	Enamel-LP	8	0.0811	0.0009	0.0800	0.0825	
	Enamel-VM	8	0.0900	0.0016	0.0881	0.0932	

VE: VITA Enamic, LU: Lava Ultimate, LP: Lava plus VM: VITA Mark II.

 Post hoc Games-Howell analysis revealed a statistically significant difference in enamel weight loss between all five study groups when tested under non-erosive conditions. However, erosive enamel wear rates were significantly different between almost all study groups except when LU and VE (0.0672 mg vs. 0.0723 mg) and LP and VM (0.0811 mg vs. 0.0900 mg) groups were compared with each other (*P* = 0.27 and *P* = 0.18, respectively). Table 3 displays these findings in greater detail.

**Table 3:** Pairwise comparison of enamel wear rates between different test groups

**First group**	**Second group**	**pH=7**		**pH=2.5**	
		**Difference in mean wear values (mg)**	***P*** ** value**	**Difference in mean wear values (mg)**	***P*** ** value**
Enamel-Enamel	Enamel-LU	-0.0033^*^	< 0.001	-0.0135^*^	< 0.001
	Enamel- VE	-0.0039^*^	< 0.001	-0.0185^*^	< 0.001
	Enamel-LP	-0.0053^*^	< 0.001	-0.0274^*^	< 0.001
	Enamel-VM	-0.0067^*^	< 0.001	-0.0362^*^	< 0.001
Enamel-LU	Enamel- VE	-.0006	0.27	-0.0051^*^	< 0.001
	Enamel-LP	-0.0021^*^	< 0.001	-0.0139^*^	< 0.001
	Enamel-VM	-0.0034^*^	0.00	-0.0228^*^	< 0.001
Enamel- VE	Enamel-LP	-0.0015^*^	0.00	-0.0088^*^	< 0.001
	Enamel-VM	-0.0028^*^	0.00	-0.0177^*^	< 0.001
Enamel-LP	Enamel-VM	-.0013	0.18	-0.0089^*^	< 0.001

VE: VITA Enamic, LU: Lava Ultimate, LP: Lava plus VM: VITA Mark II. * Statistically significant difference.

 As displayed in Table 4, the recorded mean enamel wear values in all study groups were significantly greater when the specimens were immersed in an erosive substance, i.e., acetic acid, compared to non-erosive wear conditions (*P* < 0.001 for all study groups).

**Table 4:** Comparison of erosive and non-erosive enamel wear rates in each test group

**Group**	**pH**	**Number**	**Mean (mg)**	**Standard deviation**	**Independent-samples** **t-test**
Enamel-Enamel	7	8	0.0037	0.0007	t = 51.07 *P* < 0.001
	2.5	8	0.0537	0.0027	
Enamel-LU	7	8	0.0070	0.0004	t = 90.46 P < 0.001
	2.5	8	0.0672	0.0018	
Enamel- VE	7	8	0.0076	0.0007	t = 155.76 *P* < 0.001
	2.5	8	0.0723	0.0009	
Enamel-LP	7	8	0.0091	0.0004	t = 204.67 *P* < 0.001
	2.5	8	0.0811	0.0009	
Enamel-VM	7	8	0.0104	0.0015	t = 105.16 *P* < 0.001
	2.5	8	0.0900	0.0016	

VE: VITA Enamic, LU: Lava Ultimate, LP: Lava plus VM: VITA Mark II.

## Discussion

 This study investigated the wear behavior of bovine enamel opposed to different ceramic materials under both erosive and non-erosive conditions. The results indicated significant differences in enamel wear rates based on the type of ceramic material and the pH conditions.

 The null hypothesis, which posited no difference in the wear characteristics of hybrid ceramics and conventional ceramics, was rejected. The results showed that both the type of ceramic material and the erosive conditions significantly influenced enamel wear.

 Although in vitro experimental data cannot easily be extrapolated to in vivo responses, it is imperative to determine restorative materials’ potential to damage the natural dentition. This is especially important when formulating a treatment plan for patients with occlusal disharmony and masticatory impairment.

 Under non-erosive conditions, the post hoc Games-Howell analysis revealed significant differences in enamel wear between all ceramic materials tested. The VM group exhibited the highest enamel wear, followed by LP, VE, LU, and the control enamel groups. While several researchers do not believe the hardness and wear potential of a dental ceramic to be correlated,^24-26^ these findings align with previous studies, such as Mörmann et al,^27^ which indicated that hybrid ceramics like VE and LU tend to cause less enamel wear compared to conventional ceramics due to their lower hardness values (200 MH for VE and 96 MH for LU) compared to LP (1300 MH) and VM (640 MH).

 It has been stated that VE and LU pose an elastic modulus of 21.5 and 16 GPa, respectively,^28^ approximating that of human dentin (20 GPa).^12,29^ The lower hardness and toughness properties of these ceramic materials explain why VE and LU provoke a lower degree of wear to the opposed enamel. However, since LU exhibits greater hardness values than VE, it also induces more enamel wear in abrasive and three-body wear conditions. This may be due to the difference in particle size, composition, and manufacturing procedure. According to Seghi et al,^25^ dental ceramics’ microstructure can influence their abrasive wear performance.

 Our experiments also revealed that erosive testing conditions influenced the wear behavior of hybrid ceramics, which may be rationalized by the degradation of the polymer network in acetic acid.

 When the mechanical properties of dental ceramic, such as its elastic modulus and hardness, are far above human enamel, stress concentration can cause excessive wear in the natural enamel.^30^ This justifies why the tested feldspathic (VM) and zirconia (LP) ceramics induced a greater degree of abrasive enamel wear compared to hybrid ceramics. It has been proposed that due to its high strength and toughness, zirconia ceramics can withstand surface damage under functional stress without changing frictional coefficients. This is while the opposing enamel suffers from fatigue wear, formation, and progression of small cracks.^30,31^ Aside from hardness, the surface roughness of an antagonist is also important in determining its wear-producing ability.^20^ Fine polishing after milling is a crucial step to reduce the roughness and population of surface defects. Polishing also reduces the friction between the contacting surfaces. In surfaces subjected to cyclic sliding, pre-existing defects act as stress concentrators and can become the origins of cracks.^32^

 Al-Harbi et al^33^ reported greater surface toughness and fracture resistance values for VE compared to LU ceramics. The results of the current study also demonstrated a better abrasive wear mechanism in the enamel opposed to LU blocks, which can be ascribed to its lower surface toughness and fracture resistance in comparison to VE ceramic specimens.

 These findings have important clinical implications. The higher wear resistance of hybrid ceramics suggests that they are more suitable for restorations in patients prone to enamel erosion or occlusal disharmony. The significant increase in enamel wear under erosive conditions highlights the importance of considering the patient’s dietary habits and potential for acid reflux when selecting restorative materials.

 A notable limitation of this study was the use of flat specimens, which do not replicate the occlusal anatomy of natural teeth. However, the standardized preparation of all samples ensures the reliability of the comparative results. Future studies should include anatomically shaped specimens to better simulate clinical conditions. Additionally, advanced surface analysis techniques like optical profilometry and long-term clinical studies are necessary to validate these in vitro findings and explore the impact of different surface treatments or modifications on the wear resistance of dental ceramics.

## Conclusion

 This study underscores the significant impact of ceramic material type and erosive conditions on enamel wear. Hybrid ceramics, such as VE and LU, exhibited lower enamel wear rates compared to conventional feldspathic and zirconia ceramics. These findings advocate using hybrid ceramics in clinical practice, particularly for patients at higher risk of enamel erosion. Future research should continue to explore the development of restorative materials that balance wear resistance with biocompatibility.

## Competing Interests

 The authors declare that they have no competing interests.

## Ethical Approval

 This study was approved by the Research Ethics Committee of Mashhad University of Medical Sciences (IR.MUMS.DENTISTRY.REC.1398.059).
